# Physical Localization and DNA Methylation of 45S rRNA Gene Loci in *Jatropha curcas* L.

**DOI:** 10.1371/journal.pone.0084284

**Published:** 2013-12-30

**Authors:** Zhiyun Gong, Chao Xue, Mingliang Zhang, Rui Guo, Yong Zhou, Guoxin Shi

**Affiliations:** Key Laboratory of Crop Genetics and Physiology of Jiangsu Province/Key Laboratory of Plant Functional Genomics of Ministry of Education, College of Agriculture, Yangzhou University, Yangzhou, Jiangsu, China; Oregon State University, United States of America

## Abstract

In eukaryotes, 45S rRNA genes are arranged in tandem arrays of repeat units, and not all copies are transcribed during mitosis. DNA methylation is considered to be an epigenetic marker for rDNA activation. Here, we established a clear and accurate karyogram for *Jatropha curcas* L. The chromosomal formula was found to be 2n = 2x = 22 = 12m+10sm. We found that the 45S rDNA loci were located at the termini of chromosomes 7 and 9 in *J. curcas*. The distribution of 45S rDNA has no significant difference in *J. curcas* from different sources. Based on the hybridization signal patterns, there were two forms of rDNA - dispersed and condensed. The dispersed type of signals appeared during interphase and prophase, while the condensed types appeared during different stages of mitosis. DNA methylation analysis showed that when 45S rDNA stronger signals were dispersed and connected to the nucleolus, DNA methylation levels were lower at interphase and prophase. However, when the 45S rDNA loci were condensed, especially during metaphase, they showed different forms of DNA methylation.

## Introduction

In higher eukaryotes, the ribosomal RNA genes comprising the 18S-5.8S-26S rDNA (45S rDNA) loci are generally referred to the nucleolar organizer regions (NORs). Repetitive sequence families are major components of 45S rDNA in which the repeating units are conserved [1]. The NORs can be easily identified in chromosomes by using 45S rDNA as a probe for fluorescence *in situ* hybridization (FISH) analysis [2]. Studies of repetitive sequences are useful to investigate chromosome evolution between plant species [3], [4]. The physical localization of 45S rDNA loci have been studied in maize [5], [6], wheat [7], peanut [8], rice [9], [10], pine [4], *Nymphaea* [11], the Orchidaceae [12], and many other species.

Gene expression and regulation have a close relationship with DNA or histone modification. Methylation can be an important marker for active transcription or inactivation of chromatin. In general, unmethylated or hypomethylated DNA and histone H3 lysine 4 methylation (H3K4me) are considered to be epigenetic markers for euchromatin, and are responsible for rDNA activation [13], [14], whereas hypermethylated DNA and histone H3K9 methylation are indicative of rDNA gene silencing. Inactive genes occur in chromatin that is highly methylated and more condensed than the chromatin of active genes [15]–[17]. The most widely studied epigenetic modification is DNA methylation, which mediates gene silencing [18], [19].

DNA methylation is considered to be one of the most important mechanisms for controlling gene expression, and it also probably helps to protect the plant genome from accumulating mutations [18], [20]. Methylation of cytosine at the 5′ position on the pyrimidine ring is one of the most common forms of DNA methylation, which occurs primarily at CG dinucleotides, although it has been observed in non-CG sites, such as CHG and CHH trinucleotides (where H is A, C or T) [21]. Plant nuclear DNA usually possesses high levels of 5-methylcytosine (5-MeC) [18].

Although the majority of information about DNA methylation is based on biochemical and molecular methods [22]–[24], some molecular-cytological techniques have also been developed. For example, a method based on the detection of highly methylated DNA regions after incubation with mouse anti-5-MeC antibodies was first used for analyses of DNA methylation patterns in mammalian and plant chromosomes [25]. Using this method, the distribution of 5-MeC on the somatic chromosomes of different plant species has been analysed [18], [26]–[27].

*J. curcas* (L.) is a long-lived, drought tolerant, evergreen shrub in the family Euphorbiaceae that is native to the American tropics [28]. The species is widely cultivated commercially in tropical and subtropical regions of Asia and Africa, where it grows well in marginal soils. *J. curcas* has been proposed as a potentially important energy crop, because the seeds can contain up to 40% of an inedible oil [29], [30]. *J. curcas* has become one of the most widely studied bioenergy plants for biodiesel production [31], [32]. As the cells of *J. curcas* are characterized by condensed cytoplasm and small chromosomes that are not easily dispersed, cytological studies of this species are rarely reported. Only a single report by Carvalho [33] (2008) showed the karyotype of *Jatropha*. In addition, few reports are available on the physical localization of ribosomal DNA in *Jatropha*. Therefore, the present investigations were carried out to physically localize the 45S ribosomal DNA loci on the chromosomes of *J. curcas*. In this work we describe the location of 45S rDNA loci, and show which rRNA arrays are active or inactive at different stages of mitosis.

## Materials and Methods

### Materials

*J. curcas* seeds were kindly provided by Guojiang Wu (South China Botanical Garden, Chinese Academy of Sciences, Guangzhou, China). Seeds were germinated at 30°C in soil and plantlets were grown in a greenhouse.

### Chromosome Preparation and Fluorescence in situ Hybridization (FISH)

Roots were harvested from greenhouse-grown plants. The roots were fixed in methanol-acetic acid (3∶1), and stored at −20°C until use. Root tips were macerated in 2.0% cellulose and 1.0% pectinase at 37°C for 1.5 h. Squashes were made in the fixative on a glass slide and flame dried, as described by Kurata [34].

The FISH procedure applied to mitotic chromosomes was as described in Jiang et al. [35] (1995) and Cheng et al. [36] (2001) with minor modifications. Chromosomes were denatured in 70% formamide/2× SSC at 85°C for 2 min, sequentially dehydrated in cooled 70%, 90% and 100% ethanol for 5 min, and incubated with a digoxigenin-labeled 45S rDNA probe. The probe was detected using an anti-digoxigenin-rhodamine antibody (Roche Diagnostics). Chromosomes were counterstained with 4',6-diamidino-phenylindole (DAPI) in an antifade solution (Vector Laboratories, H-1200). Chromosome images were captured with an Olympus BX60 fluorescence microscope using an Olympus DP80 CCD camera. After recording the FISH signals, the same slides were washed in PBS buffer, dehydrated in the ethanol series, and dyed by Gimesa in (1/15 M Na_2_HPO_4_∶1/15 M KH_2_PO_4_ = 1∶1) buffer.

Measurements were made on chromosomes by Olympus cellSens Dimension software. Chromosomes in five prometaphase cells were measured and the standard deviations were calculated.

### Immunodetection of 5-methyl-cytosine

Methylated cytosine residues were detected with mouse antibodies raised against 5-methylcytosine (5-MeC) (AVIVA, AMM99021) and a goat anti-mouse secondary antibody conjugated with Alexa 488 (Invitrogen; 1∶500 in 1×TNB; 0.1 M Tris-HCl, pH 7.5; 0.25 M NaCl; 0.5% blocking reagent). Before denaturation, each slide was incubated with 50 µl RNase A (1 µg/µl, sigma, R-4875) at 37°C for 1 h. Then the slides were washed in 1×PBS for three times, and air-dryed for use. After RNase A enzymolysis, the slides were denatured in 70% formamide/2×SSC for 2 min at 85°C. Incubation with primary antibody was done at 37°C for at least 4 h. The slides were washed three times in 1× PBS, and incubated with the secondary antibody under the same conditions. Chromosomes were counterstained with DAPI in Vectashield (Vector Laboratories, H-1200). After recording the immunostained signals on the Olympus BX60 fluorescent microscope, the same slides were washed in PBS buffer, dehydrated in the ethanol series, and probed with the 45S rDNA probe sequentially using the same FISH procedure described above.

## Results

### Distribution of 45S rDNA Loci in *J. curcas* from Different Sources

**Figure 1:**
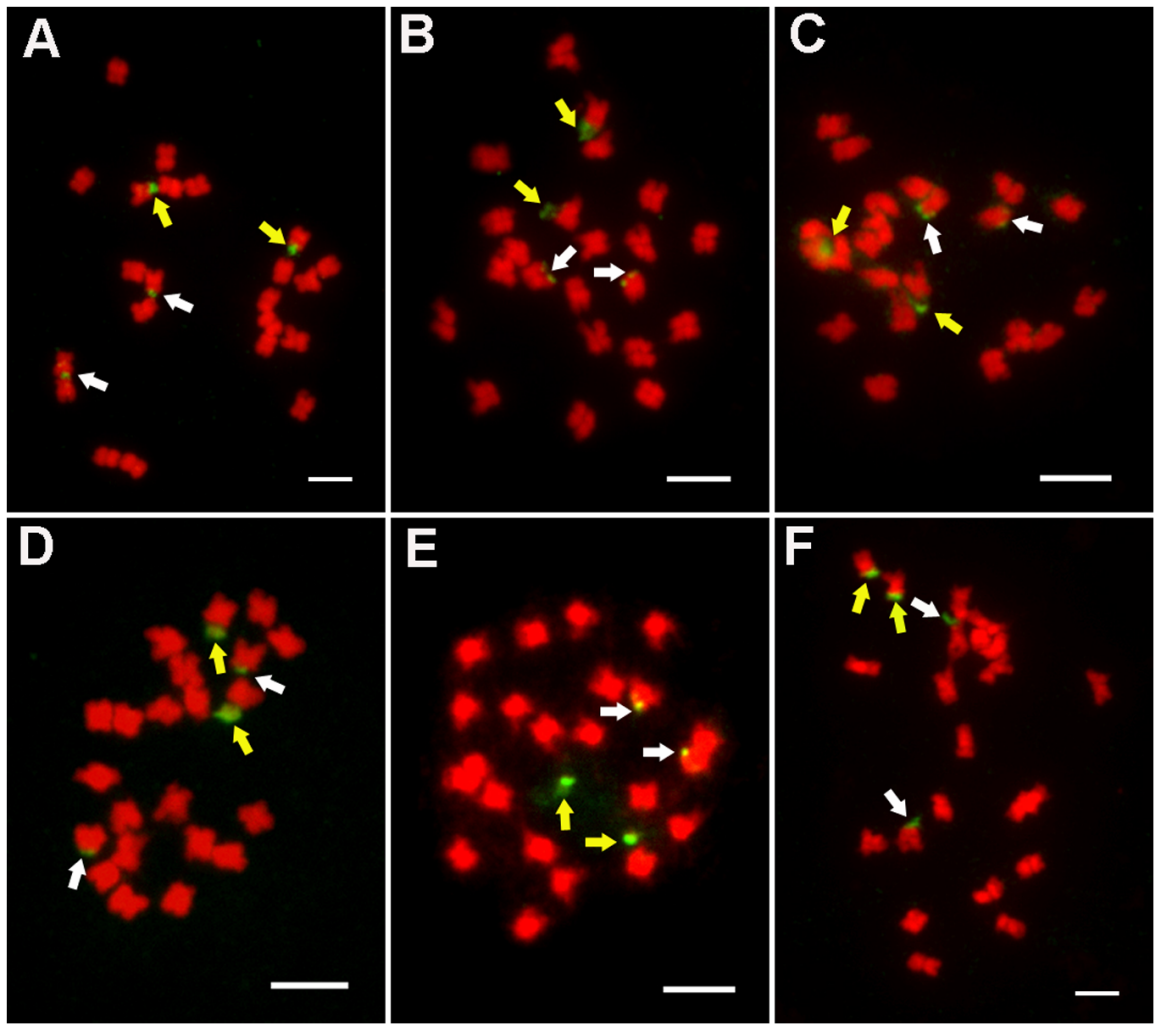
FISH analysis of *J. curcas* from different sources using 45S rDNA as a probe. **A:** Hainan; **B:** Guizhou; **C:** Guangxi; **D:** GOF70-1; **E:** GOF38-2; **F:** 17#. All chromosomes were counterstained with DAPI; all scale bars = 5 µm. The yellow arrows indicated the stronger signals and the white arrows indicated the weaker signals.

For analysis of the distribution of 45S rDNA in *Jatropha* species, we collected six materials derived from different sources and used FISH to analyze it. The result indicated that there are four loci in the cells of all the six different *Jatropha* species, located at the ends of two pairs of homologous chromosomes (Figure 1). According to the distribution of 45S rDNA in chromosomes, there is no significant difference among the *Jatropha* species derived from Hainan, Guizhou and Guangxiin China. The distribution of 45S rDNA in three different populations, GOF70-1, GOF38-2 and 17#, derived from the same area, have the similarity.

### FISH-based Karyotype Analysis of *J. curcas* using the 45S rDNA Probe

**Figure 2:**
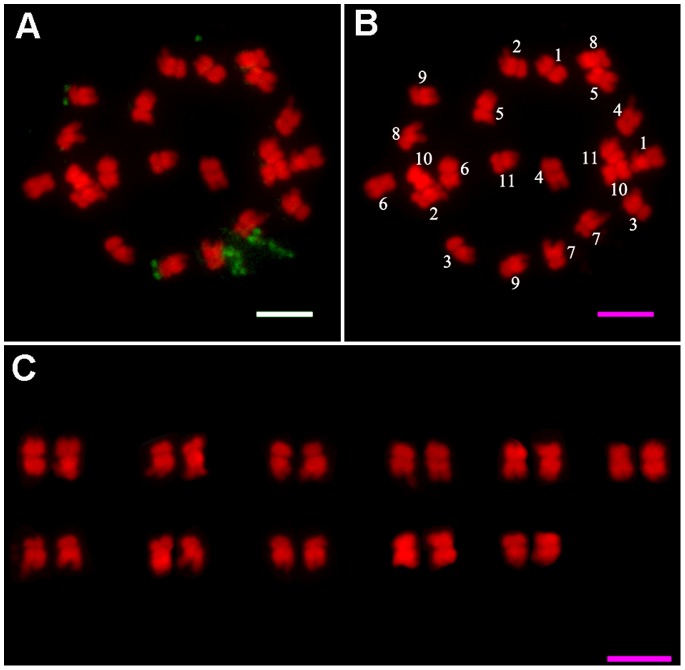
FISH and karyogram analysis of *J. curcas* prometaphase chromosomes. **A:** FISH image showing 45S rDNA probe signals on *J. curcas* metaphase chromosomes. **B:** Numbered *J. curcas* metaphase chromosomes from A. **C:** Karyogram of *J. curcas* somatic metaphase chromosomes. Scale bars = 5 µm.

Karyotype analysis has been widely applied to many plant species, but has rarely been reported for *J. curcas*. It is very difficult to obtain consistent karyograms using traditional karyotyping methods because of the relatively small chromosomes of this species. In this study, we performed high-resolution FISH for karyotype analysis of *J. curcas* using the 45S rDNA repeat sequence as a probe. Five cells with visible prometaphase chromosomes were chosen, as shown in Figure 2A; it was possible to clearly distinguish the centromeric regions of the chromosomes as well as the euchromatic and heterochromatic regions. Karyotype analysis was carried out for these five cells; chromosome lengths were expressed as absolute lengths and relative lengths, and the karyotype data were averaged as shown in Table 1. The karyotype patterns and the matching of homologous chromosomes in *J. curcas* are shown in Figure 2B and C.

**Table 1:** Morphometric data for *Jatropha curcas* chromosomes.

Chromosome	S (µm)	L (µm)	TL (µm)	r	RL	Chromosome
no.						type
1	1.63±0.14	1.77±0.12	3.34±0.13	1.09±0.07	9.99±0.25	m
2	1.08±0.08	2.44±0.23	3.32±0.26	2.27±0.28	9.94±0.82	sm
3	1.18±0.09	2.03±0.16	3.30±0.14	1.73±0.09	9.87±0.49	sm
4	1.33±0.20	1.97±0.20	3.28±0.20	1.52±0.32	9.80±0.52	m
5	1.32±0.14	1.74±0.13	3.12±0.17	1.34±0.21	9.34±0.47	m
6	1.36±0.17	1.53±0.10	3.11±0.12	1.14±0.17	9.30±0.27	m
7	1.10±0.09	2.25±0.15	3.07±0.16	2.06±0.25	9.17±0.37	sm
8	1.10±0.12	1.98±0.16	2.93±0.11	1.81±0.19	8.77±0.35	sm
9	1.03±0.10	1.97±0.21	2.80±0.22	1.93±0.25	8.37±0.60	sm
10	1.32±0.11	1.43±0.07	2.60±0.12	1.09±0.10	7.76±0.29	m
11	1.08±0.06	1.58±0.15	2.57±0.16	1.47±0.15	7.69±0.52	m

Data represents the mean ± SD of five replicates from different cells.

L: long arm length.

S: short arm length.

TL: absolute chromosome length.

RL (relative chromosome length) = 100 × TL/Total chromosome absolute length;

Arm ratio (r = L/S), length ratio between the long and short arms.

On the basis of centromere position, the arm ratio (r) was used to classify the chromosomes according to Levan et al. [48] into m, metacentric (r = 1.05−1.69); sm, submetacentric (r = 1.70−2.99); st, subtelocentric (r = 3.00−6.99); and t, telocentric (r = 7.00−39.00).

The karyotypic formula for *J. curcas* was found to be 2n = 2x = 22 = 12m+10sm. The chromosomal complement consists of six metacentric chromosome pairs (m), and five submetacentric chromosome pairs (sm). The arm ratios of the m-type chromosomes ranged from 1.52 to 1.09 with a mean of 1.27, and the arm ratios of the sm-type chromosomes showed considerable variation and ranged from 2.27 to 1.73 with a mean of 1.96. The relative lengths of the chromosomes ranged from 7.69 to 9.99, the ratio of the longest chromosome to the shortest chromosome was 1.30.

### The Location of 45S rDNA in *J. curcas*

**Figure 3:**
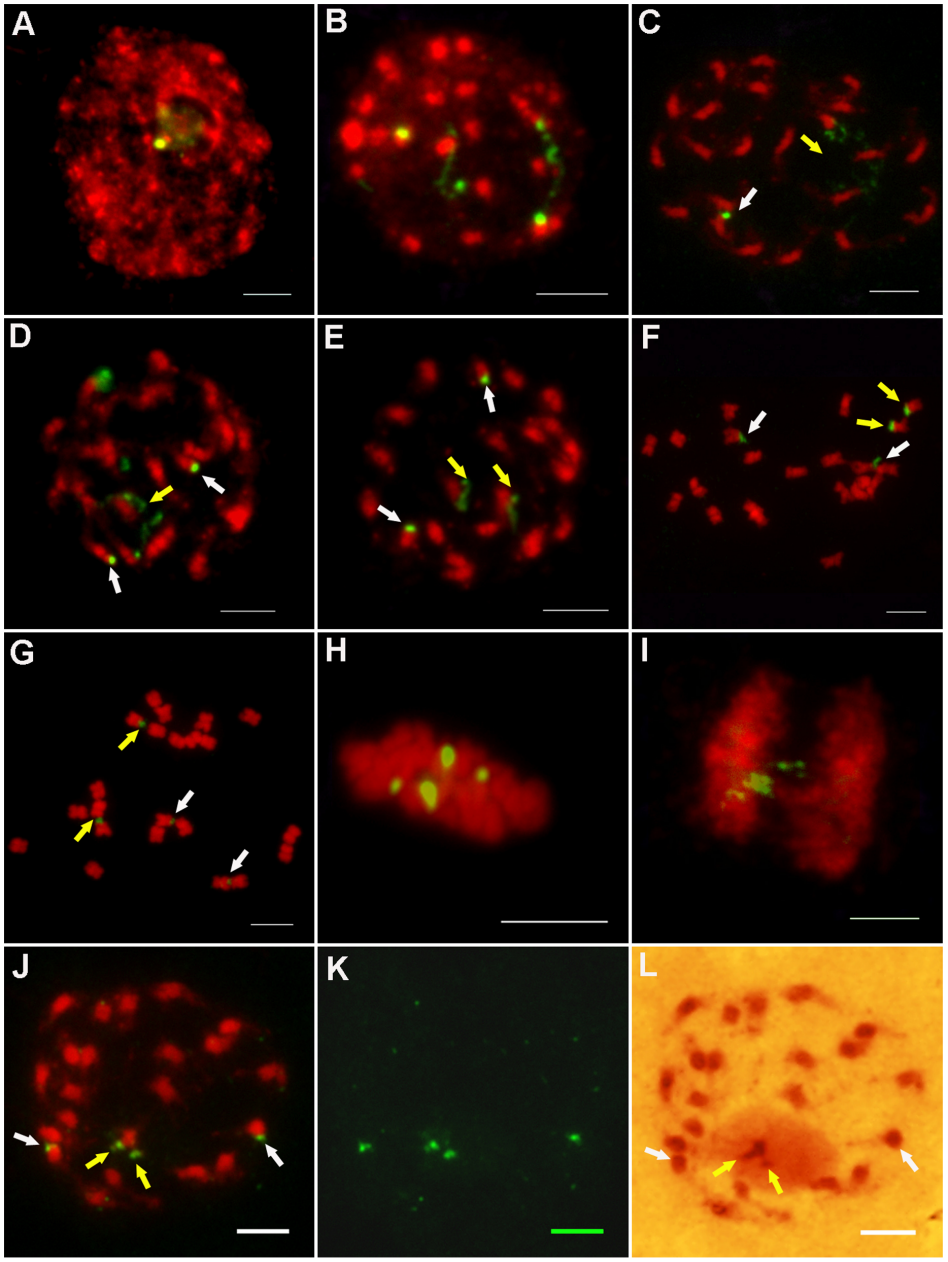
FISH analysis of *J. curcas* chromosomes using 45S rDNA as a probe at different stages of mitosis. **A–B:** Interphase; **C–E:** Prophase; **F–G:** Prometaphase; **H:** Metaphase; **I:** Anaphase. **J:** Prophase; **K:** The FISH signals of image j; **L:** The chromosome of image j dyed by Gimesa. All chromosomes were counterstained with DAPI in image A–K; all scale bars = 5 µm. The yellow arrows indicated the stronger signals and the white arrows indicated the weaker signals.

Based on the karyotype and FISH analysis, the distributions of 45S rDNA were located on the mitotic metaphase chromosomes in *J. curcas*. The results showed that two pairs of 45S rDNA signals were detected in all examined prometaphase chromosome preparations. From the karyotype analysis (Figure 2) and the summary in Table 2, we identified two pairs of 45S rDNA signals located at the ends of the short arms of chromosomes 7 and 9. One pair, on the short arm of chromosome 7, gave strong hybridization signals, while the pair on the short arm of chromosome 9 gave weaker signals. It is worth noting that the stronger signal intensities could be resolved either the condensed or dispersed forms at different stages of mitosis. At interphase, the less condensed, highly dispersed and extended hybridization signals could be seen clearly and were associated with a nucleolus (Figure 3A, B). The dispersed signals retained their extended forms from interphase to prophase (Figure 3C–E, indicated by yellow arrows). The dispersed signals became condensed at metaphase (Figure 3F–H, indicated by yellow arrows). However, the condensed signals began to decondense at anaphase (Figure 3G). In addition, the pair of weak signals remained condensed in each stage (Figure 3, indicated by white arrows). The two different signal forms indicated that ribosomal genes can appear more decondensed when they are active at interphase, are visualized as satellites or secondary constrictions at prophase, and subsequently become condensed at metaphase. The presence of inactive rDNA at metaphase could possibly indicate the presence of transcriptionally inactive rDNA. The weak signals that remained condensed at each stage of mitosis indicated that although 45S rRNA genes exist in tandem arrays, not all copies are transcribed during mitosis. In addition, rRNA is a major element of nucleolus. When rDNA was in the transcriptional state, it connected with nucleolus and the FISH signals were stronger (Figure 3J–L, indicated by yellow arrows). However, the rDNA, not being in a state of transcription, didn’t connected with nucleolus and the FISH signals were weaker (as shown in Figure 3J–L, indicated by white arrows).

**Table 2:** Summary of 45S rDNA FISH Signals and DNA methylation.

rDNA FISH	Chromosome	FISH	rDNA Methylation	Stage
Signal Intensity	No.	Forms		
Stronger	Chr. 7	Dispersed	Unmethylated	Interphase, Prophase
		Condensed	Telomeric region of rDNA	Metaphase
			methylated	
Weaker	Chr. 9	Condensed	Methylated	Interphase, Prophase
			Methylated	Metaphase
			One methylated, the other	
			unmethylated	
			Unmethylated	

In summary, the hybridization signal patterns showed that there were two forms of rDNA arrays. One type was dispersed or extended, and connected with nucleolus during interphase and prophase. At metaphase and anaphase, this signal was condensed and the nucleolus disappeared. The other type was condensed and didn’t connect with nucleolus, which appeared at each stage of mitosis.

### Distribution of 5-MeC and 45S rDNA Loci in Mitosis Cells

**Figure 4:**
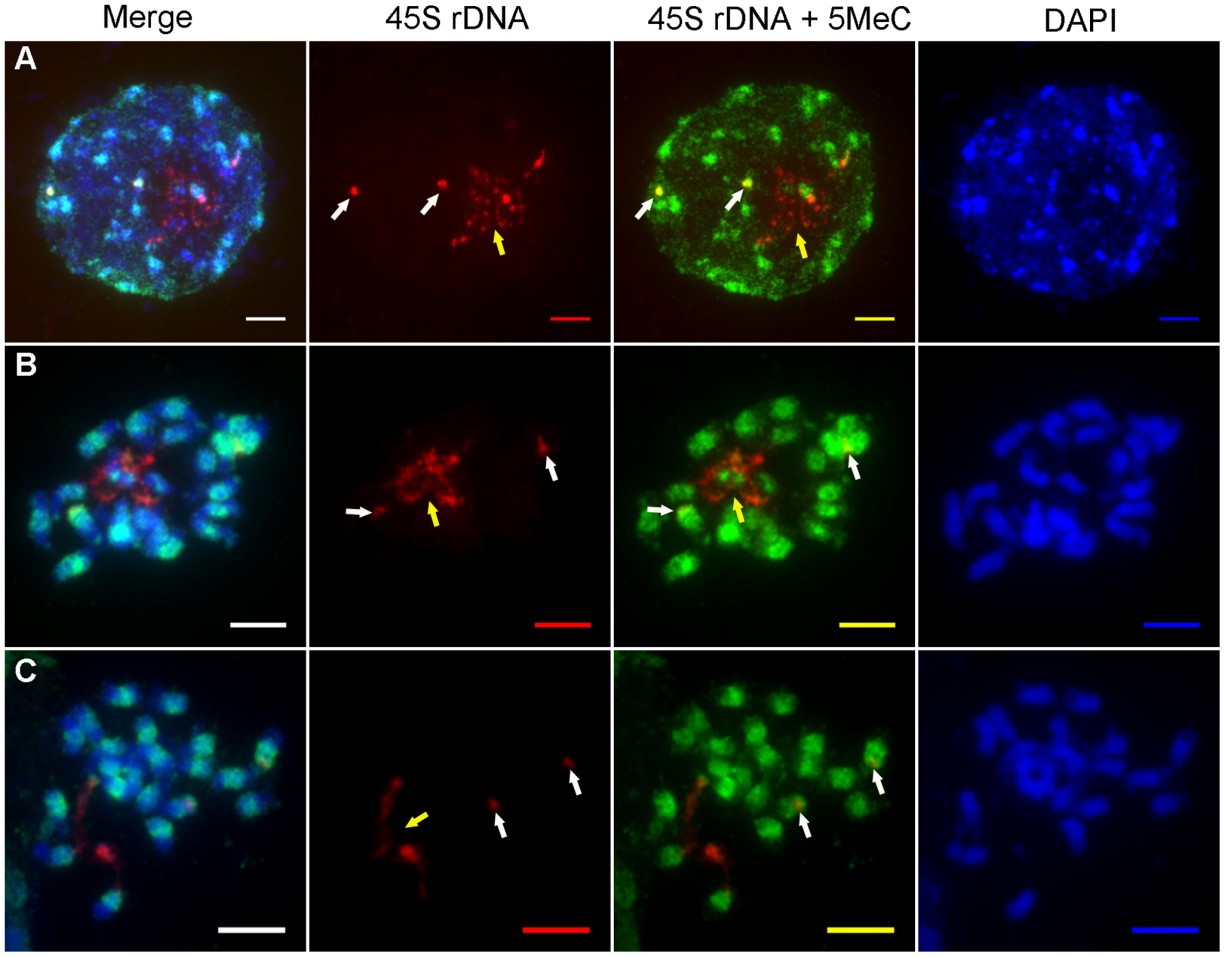
Distribution of the 5-MeC foci (green fluorescence) and 45S rDNA loci (red fluorescence) on interphase and prometaphase chromosomes *J. curcas*. **A: interphase; B–C:** prophase; All chromosomes were counterstained with DAPI. Scale bars = 5 µm. The yellow arrows indicated the stronger signals of 45S rDNA and the white arrows indicated the weaker signals of 45S rDNA.

**Figure 5:**
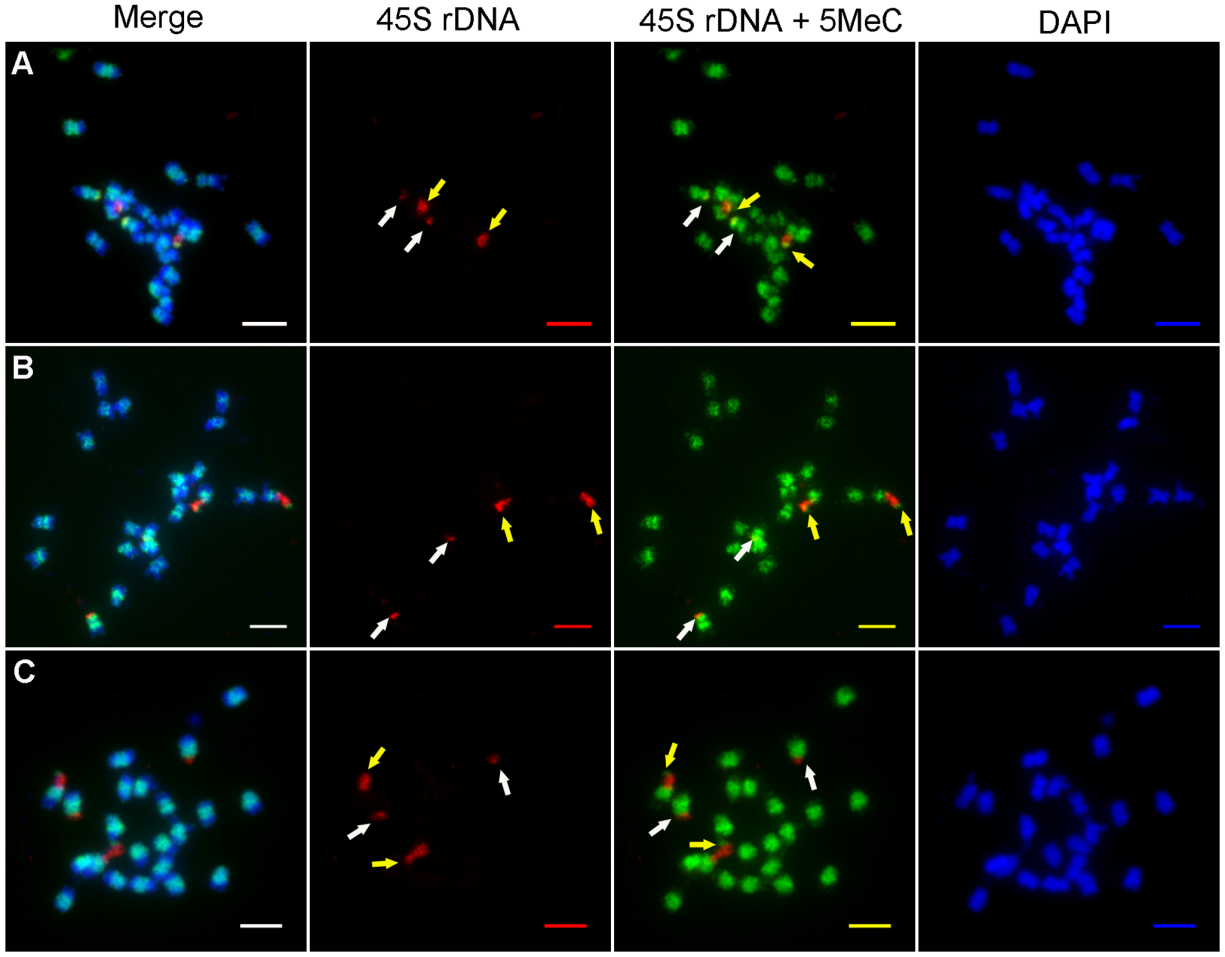
Distribution of the 5-MeC foci (green fluorescence) and 45S rDNA loci (red fluorescence) on metaphase chromosomes of *J. curcas*. **A:** In the two pairs of chromosomes, the 45S rDNA of one pair were methylated in telomeric region and the other were methylated. **(Type II)**. **B:** In the two pairs of chromosomes, the 45S rDNA of one pair were methylated in telomeric region. One of the other pair was unmethylated and the other was methylated. **(Type III)**. **C:** In the two pairs of chromosomes, the45S rDNA of one pair were methylated in telomeric region and the other were unmethylated. **(Type IV)**. All chromosomes were counterstained with DAPI. Scale bars = 5 µm. The yellow arrows indicated the stronger signals of 45S rDNA and the white arrows indicated the weaker signals of 45S rDNA.

**Figure 6:**
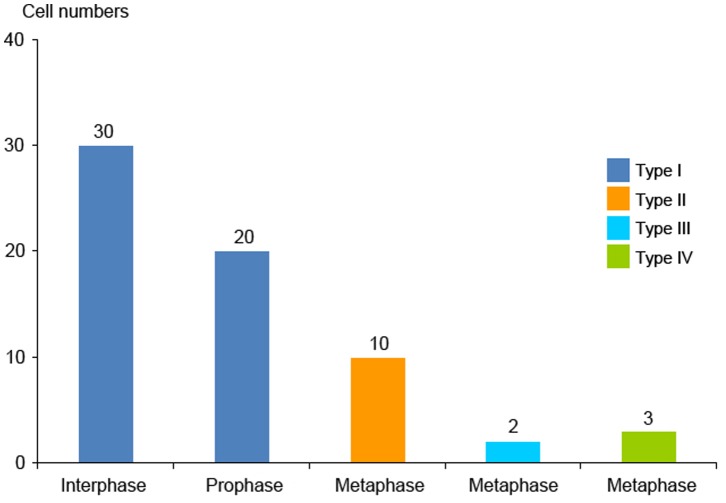
45S rDNA methylation states of the two pair chromosomes of 65 cells at different stages. **Type I:** In the two pairs of chromosomes, the 45S rDNA of one pair were unmethylated and the other were methylated. **Type II:** In the two pairs of chromosomes, the 45S rDNA of one pair were methylated in telomeric region and the other were methylated. **Type III:** In the two pairs of chromosomes, the 45S rDNA of one pair were methylated in telomeric region. One of the other pair was unmethylated and the other was methylated. **Type IV:** In the two pairs of chromosomes, the 45S rDNA of one pair were methylated in telomeric region and the other were unmethylated.

The above analysis indicated that not all copies of the 45S rRNA genes are transcribed in *J. curcas*. In plants, DNA methylation is considered to be an epigenetic marker for rDNA activation [12]. To further investigate whether the different forms of 45S rDNA have different patterns of expression, immunodetection of 5-MeC was used to analyze the DNA methylation patterns in *J. curcas* chromosomes which were incubated with RNase A. The results showed that distinct 5-MeC-containing signals were detected in chromatin in mitotic cells isolated from root meristems (Figure 4−5). We focused on analysis of the DNA methylation patterns in the nucleolar chromosomes 7 and 9, which carry the 45S rDNA loci. From interphase to metaphase, the corresponding regions of DNA methylation for 45S rDNA had four forms at different stage cells (as shown in Table 2 and Figure 6). In interphase cells, FISH analysis detected two forms of 45S rDNA signals. One form of 45S rDNA loci was dispersed (extended), while the other form appeared to be in a condensed state (Figure 4A). In the following methylation analysis, there showed low levels of methylation at the dispersed form (Figure 4A, regions indicated by yellow arrow), and the condensed form had high levels of DNA methylation (Figure 4A, indicated by white arrows) in total observed 30 cells at interphase. A similar situation was observed in total observed 20 cells at prophase (Figure 4B). In all cells of interphase and prophase, the 45S rDNA methylation belonged to the Type I in Figure 6.

When the two forms of 45S rDNA FISH signals were fully condensed at metaphase, especially the dispersed signals were separated and condensed, the corresponding regions of DNA methylation had three types in different metaphase cells (Type II, Type III, Type IV in Figure 6). In Type II, the 45S rDNA of one pair, which had stronger FISH signals, were methylated in telomeric region (Figure 5A, indicated by yellow arrows) and the other, which had weaker FISH signals, were methylated (Figure 5A, indicated by white arrows). In Type III, the 45S rDNA of one pair were methylated in telomeric region,which had stronger FISH signals (Figure 5B, indicated by yellow arrows). One of the other pair was unmethylated and the other was methylated, which had weaker FISH signals (Figure 5B, indicated by white arrows). In Type IV, the 45S rDNA of one pair, which had stronger FISH signals, were methylated in telomeric region (Figure 5C, indicated by yellow arrows) and the other which had weaker FISH signals were unmethylated (Figure 5C, indicated by white arrows). Of the 15 metaphase cells examined, the stronger signals were separated and condensed. Methylation happened in the telomeric regions, indicating that this 45S rRNA gene began to be inactive in these cells, because nucleolus disappeared in metaphase. However, even though the other 45S rDNA was in a condensed state at each stage, a portion of the rDNA genes may be unmethylated in 33% (5/15) of the cells; therefore, in metaphase, the 45S rDNA methylated signal forms were not representative of the state of rRNA gene transcription.

## Discussion

### Karyotype Analysis is Important for the Physical Localization of the 45S rDNA Loci in *J. curcas*

It is convenient to sample somatic prometaphase chromosomes in plant cytogenetic studies, and the method used for slide preparation is very well established. Using somatic prometaphase chromosomes to establish a karyogram is the conventional method of karyotype analysis, and has been reported for many species [37]–[39]. However, because of the highly condensed state of metaphase chromosomes, it is comparatively difficult to identify the centromeric regions of chromosomes and to measure the lengths of each chromosome. The banding technique is not suitable for all species [40], so the recognition and pairing of homologous chromosomes has always been a very difficult task in karyotype analysis, especially for those species having small chromosomes (like *J. curcas*). Different results can be obtained for the same species in different laboratories, which to a certain extent affects the reliability of plant karyotype analysis.

At present, there are few reports of karyotype analysis in *J. curcas*. Carvalho et al. [33] (2003) reported the traditional karyogram of *J. curcas*, but it is difficult to distinguish the homogenetic association of chromosomes with similar lengths, as well as the chromosome banding pattern due to the highly condensed chromosomes. In the present study, root somatic prometaphase chromosomes were prepared; chromosomes at this stage of mitosis are not completely condensed, and the centromeric regions could be clearly distinguished, as could the euchromatic and heterochromatic regions, further improving the reliability of karyotype analysis in *J. curcas*.

### DNA Methylation is Associated with 45S Ribosomal RNA Gene Activity during Mitosis

In plants, the 45S ribosomal RNA genes are highly conserved and are composed of tandem arrays in copy numbers ranging from hundreds to thousands [41], which makes it relatively straightforward to physically localize the loci on the chromosomes by FISH analysis. In most species, 45S rDNA loci are located at the ends of chromosomes. However, they can be located in the precentromeric regions of the chromosomes in a few species [12], [42]. In present study, the 45S rDNA loci were found to be located at the termini of two pairs of chromosomes in *J. curcas*, which is consistent with observations in many other plant species. Based on the hybridization patterns, there are two forms of rDNA signals in *J. curcas*: one type was dispersed or extended, and the other was condensed. The dispersed signals appeared during interphase and prophase, while the condensed type appeared at different stages in mitosis. The extended signals evidently represent the transcriptionally active rRNA genes which connected with nucleolus, and the condensed signals indicate rDNA that is in a state of inactivation [12], [43]. In addition, the rRNA genes comprise the nucleolar organizer regions (NORs), which are the sites of ribosome synthesis and organize active rRNA genes at interphase [41], [44]. Although rDNA sequences are arranged as tandem repeats, not all are expressed [12]. In this study, the 45S rDNA signals were extended, which indicated that the rRNA genes were active and connected with nucleolus at interphase and prophase in *J. curcas*. However, the FISH signals were not completely extended, as rDNA was arranged in repetitive sequences, only some of which were expressed [12], [18].

Within the tandemly arranged rDNA units, active and inactive rRNA genes can be differentiated by epigenetic markers, such as DNA methylation [12]. CG methylation is sufficient for transposable element inactivation [19], [45]. When rDNA genes are active and expressed, DNA methylation levels are low or undetectable [18], . In addition, highly methylated genes are heterochromatic or inactive [19]. However, DNA methylation alone was unlikely to explain nucleolar dominance in all organisms [13], [14], In this study, when the 45S rDNA signals were dispersed, DNA methylation levels were lower at interphase and prophase, in which nucleolus didn’t disappear. This suggested the 45S rDNA FISH signal forms represented the transcriptional state of the rRNA gene loci at interphase and prophase, when nucleolus didn’t disappear. However, when 45S rDNA loci were condensed at metaphase and nucleolus disappeared, DNA methylation levels differed between cells at metaphase. This suggested that the 45S rDNA FISH signal forms did not represent the transcriptional state of the rRNA gene loci at metaphase, when nucleolus disappeared.
